# Who is the main caregiver of the mother during the doing-the-month: is there an association with postpartum depression?

**DOI:** 10.1186/s12888-021-03203-4

**Published:** 2021-05-25

**Authors:** Ke Peng, Lin Zhou, Xiaoying Liu, Menglu Ouyang, Jessica Gong, Yuanyuan Wang, Yu Shi, Jiani Chen, Yichong Li, Mingfan Sun, Yueyun Wang, Wei Lin, Shixin Yuan, Bo Wu, Lei Si

**Affiliations:** 1 National Clinical Research Center for Cardiovascular Diseases, Shenzhen, Fuwai Hospital Chinese Academy of Medical Sciences, Shenzhen, Shenzhen, China; 2 Affiliated Shenzhen Maternity and Child Healthcare Hospital, Southern Medical University, Shenzhen, China; 3 Shenzhen Centre for Disease Control and Prevention, Shenzhen, China; 4 School of Medical Sciences, Faculty of Medicine and Health, The University of Sydney, Sydney, Australia; 5 The George Institute for Global Health, Faculty of Medicine, University of New South Wales, Sydney, Australia; 6 Division of Psychology, Faculty of Health and Life Sciences, De Montfort University, Leicester, UK; 7 University International College, Macau University of Science and Technology, Macau, China; 8 School of Health Policy & Management, Nanjing Medical University, Nanjing, China

**Keywords:** Doing-the-month, Postpartum depression, Caregiver, China

## Abstract

**Background:**

To examine the relationship between the main caregiver during the “doing-the-month” (a traditional Chinese practice which a mother is confined at home for 1 month after giving birth) and the risk of postpartum depression (PPD) in postnatal women.

**Methods:**

Participants were postnatal women stayed in hospital and women who attended the hospital for postpartum examination, at 14–60 days after delivery from November 1, 2013 to December 30, 2013. Postpartum depression status was assessed using the Edinburgh Postnatal Depression Scale. Univariate and multivariable logistic regressions were used to identify the associations between the main caregiver during “doing-the-month” and the risk of PPD in postnatal women.

**Results:**

One thousand three hundred twenty-five postnatal women with a mean (SD) age of 28 (4.58) years were included in the analyses. The median score (IQR) of PPD was 6.0 (2, 10) and the prevalence of PPD was 27%. Of these postnatal women, 44.5% were cared by their mother-in-law in the first month after delivery, 36.3% cared by own mother, 11.1% by “yuesao” or “maternity matron” and 8.1% by other relatives. No association was found between the main caregivers and the risk of PPD after multiple adjustments.

**Conclusions:**

Although no association between the main caregivers and the risk of PPD during doing-the-month was identified, considering the increasing prevalence of PPD in Chinese women, and the contradictions between traditional culture and latest scientific evidence for some of the doing-the-month practices, public health interventions aim to increase the awareness of PPD among caregivers and family members are warranted.

## Introduction

Postpartum depression (PPD) is a debilitating but treatable mental disorder that occurs after childbirth [1]. Symptoms of PPD include sleep disturbance, anxiety, irritability, feeling of overwhelmed and obsessional preoccupation with the baby’s health and feeding [1]. Suicide intention and harm to baby have also been reported [2]. PPD affects one in nine new mothers after childbirth, with prevalence ranging from 10 to 15% worldwide, and this is even higher in low- and middle- income countries [2]. In China, the prevalence of PPD was 15.5% [3].

Past history of anxiety and untreated depression during pregnancy is the strongest risk factor of PPD [4]. In addition to reduced level of reproductive hormones, social determinants including social-economic levels, marital status and other factors such as sleep deprivation, infant’s sex and being primiparous were also well-recognised risk factors associated with PPD [5–7]. Moreover, social support is the key factor involved at the onset of depression and anxiety disorders [3]. Lack of support from spouse or family, and unsatisfactory marital relationship are also related to increased risk of PPD [3]. The availability and perception of social support might also be related to the development of PPD [8].

In China, “zuoyuezi”, or ‘doing-the-month’, a traditional practice for postpartum care, which mothers stay at home for a month immediately after childbirth, is a form of social support for the mothers in Chinese society [8, 9]. According to the theory of Traditional Chinese Medicine, there is an emphasis on balancing the yin and yang to maintain health. From the theory, the balance of yin and yang in prenatal women can be disrupted by labour. The rules of doing-the-month require the new mother to stay at home, and to avoid any physical labour, and anything cold for 1 month, which are believed to play important roles in regaining the balance of yin and yang, and to avoid unwell and misfortune [10].

Previous studies suggested that the traditional practice may prevent PPD in postnatal women, which may be due to the social supports and networks provided to the mothers during doing-the-month [11, 12]. The caregivers, who assist domestic duties to maintain the daily life of new mothers during doing-the-month, are often the women’s mother, mother-in-law, yuesao (a maternity matron who specializes in caring for mother and newborn infant) and/or family relatives [3]. Previous studies showed that postpartum care provided by women’s own mother exhibited fewer depressive symptoms. In contrast, mother-in-law as the main caregiver was a risk factor for PPD [3]. In many Asian countries, conflict between daughter- and mother-in-law is an essential cause of household distress [13]. A population-based study in Hong Kong showed that conflict with mother-in-law independently predicted the occurrence of PPD [14]. Power of decision-making at home and increased social support have been considered as the most important factors to promote women’s reproductive health [15]. However, caregiver’s identity was not found as an important risk factor of PPD in previous studies. In contrast, mother’s education, socioeconomic status, multiparity, history of depression, pregestational diabetes and negative birth experience were significantly associated with PPD [16, 17]. A study showed that caregiving was mediated by lower level of parental self-efficacy and lower marital satisfaction, and this study indicated that caregivers were not significant to PPD [18]. Therefore, the association between the role of caregivers and the risk of PPD for new mothers still remained unclear. In this study, we aim to investigate the relationship between the main caregivers of the mother during doing-the-month and compare the association between each type of caregiver and risk of PPD.

## Methods

### Participants

The present study used data from a pregnant and puerperal women mental health project conducted in Shenzhen City, China in 2013. The study was a single centre cross-sectional study carried out in the Shenzhen Maternity and Child Health Hospital. All postnatal women were recruited from the hospital where they delivered the baby and the hospital where postnatal women attended postpartum examination at 14–60 days after delivery from November 1, 2013 to December 30, 2013. A total of 1325 participants were included for the final analysis (*n* = 64 participants were excluded due to missing information regarding PPD).

### Data collection

A study-specific questionnaire solicited information on demographic characteristics, obstetric information, pregnancy stress and socio-cultural factors was administered by nurses from the Shenzhen Maternity and Child Health Hospital. The study nurses were trained to follow the study protocol prior to study commencement. Ethics approval was obtained from the ethics committee of the Shenzhen Maternity and Child Health Hospital. A hard copy of the consent form was provided to each study participant.

### Measurements

#### Assessment of covariates

The following demographic information was solicited: age, household registration (native or immigrant), monthly household income (categorized as <=10,000 Chinese yuan and > 10,000 Chinese yuan), living situation (only with husband, with puerperal women’s parents-in-law, with puerperal women’s parents), education level (categorised as junior high school or lower, high school or above), occupation (housewife or others) and medical insurance. Self-reported smoking and drinking behaviours were collected. The main caregiver of the mother in the first month after delivery (mother-in-law, mother, yuesao, other relatives) and obstetric information on parity (primiparous or parous), mode of delivery (vaginal or caesarean section), infant’s sex, and infant’s birth weight were also collected from the participants.

### Stress during pregnancy

Stress during pregnancy was assessed by the Chinese version of the Pregnancy Stress Rating Scale (PSRS), which is a validated instrument among Chinese pregnant women [19]. The stress level during pregnancy was estimated using the sum of scores (ranging from 0 to 90), with a higher score indicating a higher stress level.

### Social support

Social support was assessed by the Chinese version of the Social Support Rating Scale (SSRS) which has been validated in the Chinese population [19]. The sum of scores (ranging from 0 to 90) was used to indicate the level of social support received by participants, with a higher score indicating better social support.

### Postpartum depression

PPD was assessed using the Chinese version of the Edinburgh Postnatal Depression Scale (EPDS), which has been validated in Chinese puerperal women [20, 21]. The PPD was evaluated using the sum of scores (ranging from 0 to 30), with a higher score indicating a higher PPD level. The cut-off score to detect depression was defined as 9/10, which was found to be a reliable cut-off among Chinese women [21].

### Statistical analysis

Descriptive statistics were used to summarize the characteristics of study participants. Univariate logistic regression was performed to assess the association between putative factors and PPD risk. The risk factors included in the analyses were age, education level, household registration, household income, occupation, medical insurance, smoking, drinking, parity, mode of delivery, living situation, main caregiver of puerperal women, social support and stress during pregnancy. Variables with a *p*-value < 0.5 in the univariate analyses were then selected into multivariate logistic regression models. For multivariable logistic regression models, model 1 examined the association between the main caregiver of the mother and the risk of PPD with adjustment for demographic characteristics (household income and occupation), smoking and drinking. Model 2 additionally adjusted for parity, delivery mode and infant’s weight. The full model (model 3) further adjusted for living situation, social support and stress during pregnancy. A sensitivity analysis was conducted to investigate the association between the main caregiver of the mother and the trend of PPD score by performing multivariable linear regression models. Significant level was defined as *p* value < 0.5 and all analyses were performed using the SAS 9.4 (SAS institute, Cary, NC, USA).

## Results

A total of 1325 puerperal women were included in the study. The mean (SD) age was 28 (4.58) years and the median score of PPD was 6.0 (2, 10). 26.6% of the study participants were identified with PPD. Women who had lower household income, smoked and drank regularly, primiparous, lived with parents-in-law, had less social support and with a higher stress level during pregnancy were more likely to suffer from PPD. Nearly half (44.5%) of the participants were cared by their mother-in-law during the doing-the-month, followed by 36.3% cared by their own mother, 11.1% by yuesao and 8.1% by other relatives. The characteristics of the study participants are shown in Table 1.

**Table 1:** Baseline characteristics of the study participants

Variable	*N* = 1325
Age^a^	28.0 (4.58)
Education level	
Junior high school or below	327 (24.9%)
High school or above	897 (75.1%)
Household registration	
Native	373 (29.4%)
Immigrant	898 (70.6%)
Household income (Yuan/month)	
≤10,000	849 (67.4%)
> 10,000	411 (32.6%)
Occupation	
Employed	952 (73.5%)
Housewife	343 (26.5%)
Medical insurance	
No	568 (44.8%)
Yes	700 (55.2%)
Smoking	
No	1294 (97.8%)
Yes	29 (2.2%)
Drinking	
No	1285 (97.3%)
Yes	36 (2.7%)
Parity	
Primiparous	250 (19.7%)
Parous	1021 (80.3%)
Mode of delivery	
Vaginal delivery	784 (61.6%)
Caesarean section	489 (38.4%)
Infant’s sex	
Female	375 (44.4%)
Male	470 (55.6%)
Infant’s weight^a^	3.4 (0.47)
Living situation	
Living only with husband	589 (45.9%)
Living with puerperal women’s parents-in-law	254 (19.8%)
Living with puerperal women’s parents	440 (34.3%)
Caregiver of puerperal women	
Mother-in-law	590 (44.5%)
Mother	481 (36.3%)
Yuesao	147 (11.1%)
Other relatives	107 (8.1%)
Social support^a^	41.7 (6.98)
Stress during pregnancy^a^	13.0 (5,26)
EPDS score^b^	6.0 (2,10)

Values are ^a^means (SD) or ^b^median (25th and 75th percentiles) for continuous variables and percentages for categorical variables

Table 2 shows the results of the univariate analysis.

**Table 2:** Univariate analysis

Variables	Depressed *n* = 352(26.6%)	Non-depressed *n* = 973 (73.4%)	OR	95% CI	*P*
Age	27.9 (4.6)	28.0 (4.6)	0.994	0.968–1.021	0.67
Caregiver of puerperal women					
Mother	117 (33.2)	364 (37.4)	ref		
Yuesao	41 (11.7)	106 (10.9)	1.203	0.794–1.825	0.3835
Mother-in-law	169 (48.0)	421 (43.3)	1.249	0.949–1.643	0.1123
Other relatives	25 (7.1)	82 (8.4)	0.949	0.579–1.554	0.8339
Education level					
≥High school	262 (74.9)	725 (75.2))			
< High school	88 (25.1)	239 (24.8)	1.019	0.768–1.351	0.8965
Household registration					
Native	102 (30.3)	271 (29.0)			
Immigrant	235 (69.7)	663 (71.0)	0.942	0.717–1.236	0.6652
Household income (yuan/month)					
≥10,000	84 (24.9)	327 (35.4))			
< 10,000	253 (75.1)	596 (64.6)	1.652	1.247–2.189	**0.0005**
Occupation					
Employed	259 (75.3)	693 (72.9)			
Housewife	85 (24.7)	258 (27.1)	0.882	0.664–1.171	0.3836
Smoking					
No	339 (96.3)	955 (98.4)			
Yes	13 (3.7)	16 (1.6)	0.437	0.208–0.917	**0.0287**
Drinking					
No	336 (95.4)	949 (97.9)			
Yes	16 (4.6)	20 (2.1)	0.442	0.227–0.864	**0.0169**
Medical insurance					
Yes	185 (54.7)	515 (55.4)			
No	153 (45.3)	415 (44.6)	1.026	0.799–1.318	0.8387
Parity					
Primiparous	81 (24.2)	169 (18.1)			
Parous	254 (75.8)	767 (81.9)	0.691	0.512–0.933	**0.0159**
Delivery mode					
Vaginal delivery	218 (65.5)	566 (60.2)			
Caesarean section	115 (34.5)	374 (39.8)	0.798	0.615–1.036	0.0907
Infant’s sex					
Male	133 (55.4)	337 (55.7)			
Female	107 (44.6)	268 (44.3)	1.012	0.749–1.367	0.9399
Infant’s weight	3.3 (0.5)	3.4 (0.5)	0.821	0.631–1.070	0.1444
Living situation					
Living only with husband	147 (43.2)	442 (46.9)	ref		
Living with puerperal women’s parents	59 (17.4)	192 (20.7)	0.910	0.644–1.286	0.5920
Living with puerperal women’s parents-in-law	134 (39.4)	306 (32.5)	1.317	0.999–1.735	0.0505
Social support	39.2 (7.4)	42.7 (6.6)	0.930	0.913–0.948	**<.0001**
Stress during pregnancy	25.7 (15.2)	10 (4.20)	1.060	1.050–1.071	**<.0001**

Values are means (SD) for continuous variables and percentages for categorical variables

Table 3 shows the results of the multivariate analysis. Overall, compared to those whose main caregiver was her own mother, the odds of PPD compared to those whose main caregivers were yuesao, mother-in-law and other relatives were 1.16 (95% CI: 0.74–1.81), 1.23 (95% CI: 0.92–1.65) and 0.86 (95% CI: 0.51–1.47), respectively. The associations attenuated slightly after adjusting for covariates.

**Table 3:** Multivariate analysis

	Caregiver of puerperal women	OR	95% CI	*P*
Model 1	Mother	ref		
	Yuesao	1.156	0.739–1.810	0.5248
	Mother-in-law	1.233	0.921–1.650	0.1596
	Other relatives	0.861	0.505–1.467	0.5815
Model 2	Mother	ref		
	Yuesao	1.161	0.723–1.864	0.5369
	Mother-in-law	1.249	0.918–1.700	0.1572
	Other relatives	0.849	0.473–1.524	0.5836
Model 3	Mother	ref		
	Yuesao	1.053	0.607–1.824	0.8548
	Mother-in-law	0.884	0.597–1.310	0.5402
	Other relatives	0.695	0.347–1.391	0.3039

Model 1: Household income, occupation, smoking and drinking;

Model 2: Model 1 + parity, delivery mode and infant’s weight;

Model 3: Model 2 + living situation, social support and stress during pregnancy

Table 4 shows the results of the sensitivity analysis. Compared with those whose main caregiver was their own mother, being cared by yuesao and mother-in-law had a 17.4% (*p* = 0.01) and 10.3% (*p* = 0.04) higher score of PPD after adjusting for covariates including household income, occupation, smoking, drinking, parity, delivery mode, infant’s weight, living situation, social support and stress during pregnancy. No differences in PPD occurrence in the new mother cared by her own mother and other relatives were found (*p* = 0.69).

**Table 4:** Sensitivity analysis, multivariate linear regression analysis

		Unstandardised coefficient		Standardized coefficient	t	*p*
		B	Std. Error	Beta		
Sensitivity analysis	Caregiver of puerperal women					
	Mother	ref	ref	ref	ref	ref
	Yuesao	0.16050	0.06557	0.07539	2.45	0.0145
	Mother-in-law	0.09881	0.04682	0.07219	2.11	0.0351
	Other relatives	−0.03139	0.07857	−0.01217	−0.40	0.6896

Model: adjusted to occupation, household income, smoking and drinking, living situation and social support, parity and stress during pregnancy

## Discussion

In this study population, around 27% of postpartum women were identified with PPD. The present study did not find a significant relationship between the main caregiver during doing-the-month and the presence of postpartum depression.

Doing-the-month is a set of traditional postpartum practices that has been practiced with a long history in China [10]. Although some of practices of doing-the-month have been abandoned, most of the Chinese women are brought up to believe that adherence to these practices of doing-the-month is important to their postnatal recovery and quality of life after childbirth [22]. Before yuesao became popular, the common practice of the doing-the-month came from the older generation including mother, mother-in-law or relatives (e.g. grandmother) [8]. Mother, mother-in-law and yuesao have similar perspectives and experiences in practicing doing-the-month. We speculated that different generations follow similar practices and mothers of newborns tend to follow these rules, as such, the person who provides doing-the-month guidance is not a contributor of postpartum depression. However, we note that this finding was not consistent with other studies from Beijing [3], Hong Kong [23] and Taiwan [24], which reported that the conflict between postnatal women and their mother-in-law during doing-the-month was a risk factor for postpartum depression. Indeed, conflict with mother-in-law is believed to be a significant contributor to depression and related events among married Chinese women [25, 26]. It was suggested that the conflict between mother-in-law and daughter-in-law may offset the potential benefits of family support [27]. Interestingly, we have previously reported that living with mother-in-law was associated with a higher risk of PPD in this population [28]. The findings in the same population reflected the conflicts between the traditional Chinese culture of obeying older generations, and modern values of independence and autonomy [23, 28].

Although a non-significant association between the main caregiver and the risk of PPD was identified in the present study, social support was found to be strongly associated with PPD in the univariate analysis. Consistently, a prospective cohort study conducted in China reported a reverse association between prenatal and postnatal social support and risk of PPD [29]. Meanwhile, another prospective study also found a lower perceived social support to be associated with PPD symptoms [30]. Other risk factors identified by the previous studies included smoking, drinking, stress during pregnancy, household income and parity were consistently found in our study [7, 31–35].

Regardless who the person is providing guidance or support for doing-the-month, evidence on doing-the-month itself and the development of PPD remains inconclusive [36]. Back in 1980s, PPD was not a major concern in the Chinese society because the prevalence was low (approximately 1.0–2.4%) [37, 38]. Doing-the-month practice was regarded as a protective factor for PPD because it provides social support to the mothers [39]. A recent study has reported that the prevalence of perinatal depression was 17–20% in China [40], or may be greater than 25%, depending on the criteria and timing for diagnosis, levels of urbanization and population characteristics [41, 42]. It is clear that the prevalence of PPD in China has increased in the past decades.

Apart from the increasing prevalence of PPD, advances in science and medicine also challenge the Chinese traditional culture, including some of the doing-the-month practice. For example, physical activity is not encouraged during doing-the-month [23]; in contrast, it has been demonstrated that physical activity may reduce the risk of excessive weight gain during pregnancy, gestational diabetes and PPD [43]. While who is the caregiver during doing-the-month has no association with the risk of PPD, caregiver’s role as an important component in social support in preventing new mother’s PPD, the increasing trend of PPD, as well as other mental health issues should not be overlooked.

The strengths of the current study included large sample size, and there was ample information on other potential risk factors of postpartum depression. This allows the inclusion of possible confounders in the statistical analysis to produce stable results.

Several limitations should be acknowledged. Firstly, due to the cross-sectional design, no causal effect can be concluded from our findings. Secondly, participants were drawn from single study-center, which limited the generalizability of the findings. Furthermore, the current survey was conducted at 14–60 days after delivery, as such, the association with long-term outcome such as major depression, cannot be determined. Although the conflict with mother-in-law was not a significant factor of stress during doing-the-month in this population, it is not possible to estimate the long-term effect of these risk factors on advanced outcomes such as major depression and suicide events. Future studies investigating the relationship between traditional cultural factors and long-term mental health among Chinese women are needed. Residual confounding may also be possible, due to the lack of information on spousal relationship, autonomy or decision making and other unmeasured factors related to PPD.

In summary, our study does not support an association between the main caregivers of the mother and the risk of PPD during the doing-the-month. Considering the growing prevalence of PPD in Chinese women, and the contradictions between traditional culture and latest scientific evidence for some of the doing-the-month practices, public health interventions which aim to increase the awareness of PPD among caregivers and family members are warranted.

## Data Availability

The datasets generated during and/or analysed during the current study are available from the corresponding author on reasonable request.

## Abbreviations

PPD: Postpartum Depression
PSRS: Pregnancy Stress Rating Scale
SSRS: Social Support Rating Scale
EPDS: Edinburgh Postnatal Depression Scale
SD: Standard Deviation
IQR: Interquartile Range
